# Examining the relation between the subjective and objective social status with health reported needs and health-seeking behaviour in Dande, Angola

**DOI:** 10.1186/s12889-021-11003-4

**Published:** 2021-05-25

**Authors:** Edite Vila Nova Rosário, Milton Severo, Diogo Francisco, Miguel Brito, Diogo Costa

**Affiliations:** 1 CISA – Centro de Investigação em Saúde de Angola (Health Research Centre of Angola), Hospital Geral do Caxito, Rua Direita, Caxito, Angola; 2 Instituto de Saúde Pública da Universidade do Porto (ISPUP), Oporto, Portugal; 3 Health and Technology Research Centre (H&TRC), Escola Superior de Tecnologia da Saúde de Lisboa, Instituto Politécnico de Lisboa, Lisbon, Portugal; 4 Department of Population Medicine and Health Services Research, School of Public Health, Bielefeld University, Bielefeld, Germany

**Keywords:** Subjective social status, MacArthur scale of subjective social status, Socioeconomic position, Angola, Health and demographic surveillance system, Self-reported health, Health-seeking behaviour

## Abstract

**Background:**

Assessing subjective social status (SSS) may be easily accommodated in the context of a Health and Demographic Surveillance System (HDSS). To our knowledge, no prior studies have examined the association of SSS and health in Angola. Subjective socioeconomic measures may provide a rapid assessment of a relevant social status construct, important for studying health inequalities. In this study, we addressed social determinants of health by examining the relationship between the subjective and objective social status, reported health and healthcare-seeking behaviour.

**Methods:**

This research results from a cross-sectional study performed during 2015 in the Dande HDSS, in Angola. We tested the application of the MacArthur scale as a measure of SSS in a developing setting, in a sample of 12,246 households. First, we investigated its relation to objective socioeconomic indicators, and then we explored how subjective and objective social status associate with health reported needs and health-seeking behaviour of the surveyed population.

Chi-square, ANOVA tests, and Receiver Operating Characteristics (ROC) Curves analysis were computed for testing relationships between subjective status ladder quartiles, sociodemographic and household characteristics. Logistic regression was used to examine the influence of subjective perception of status in self-reported health and health-seeking behaviour.

**Results:**

Our findings suggest that the SSS follows a gradient distribution obtained with more objective socioeconomic indicators. Additionally, we found that subjective perception of status influence health needs reporting and health-seeking behaviour and its significant effect remained after controlling for the objective socioeconomic markers. Individuals standing in the second quartile of the social ladder have more odds of reporting illness and those in the highest quartiles of the ladder were twice more likely (OR = 2.23, 95% CI = 1.52–3.26) to seek help from formal health services than those at the bottom of the ladder.

**Conclusions:**

The MacArthur Scale is a valuable tool to measure SSS in the Dande HDSS, relevant for studying socioeconomic disparities and health inequalities. It is also an easier alternative to traditional measures such as income, usually difficult to measure in developing settings. The social perception of status should be considered as a complement with objective indicators when exploring social determinants of health.

**Supplementary Information:**

The online version contains supplementary material available at 10.1186/s12889-021-11003-4.

## Background

Social and economic conditions and their effects on people’s lives determine their risk of illness and their action to prevent or to treat it when it occurs [1, 2]. Unequal distribution of resources and social goods leads to different degrees of economic, political, social, and cultural advantage among groups, which may affect individuals’ health [3]. Among those factors, socioeconomic status (SES), a central feature of all societies’ social structure [4], has received remarkable attention on public health and epidemiological research [5, 6].

Socioeconomic status is a theoretical construct encompassing individual, household, and/or community access to resources [7]. It measures an individual’s economic (e.g., material goods and assets) and sociological (prestige within a community) standing [8], and has been commonly considered an important predictor of health. Indeed, in health science literature, it is well established that SES is a compelling determinant of morbidity, mortality, and self-rated health [9–12].

Wealth indexes, housing conditions, education, income, and occupation, have been widely used and became conventional measures of objective socioeconomic status (OSS) [3, 5, 13, 14]. The latter indicators are the most frequently used to operationalise SES and proved to be very useful in describing and evaluating health inequalities [3, 4, 15]. Generally, evidence shows that disadvantaged and less educated populations have poorer health than their better-off counterparts, have lower coverage of preventative health interventions, and lower life expectancy, among other health outcomes [1, 16].

The consistent finding of this gradient in health disparities throughout the social hierarchy, and not only below a certain threshold of poverty, suggests that, beyond individuals’ material circumstances, dimensions as health behaviours, psychological factors and perceptions of social ordering also intervene in these associations [10, 17]. Individuals’ perception of their social position, combined with associated emotions resulting from their beliefs, such as stress, self-esteem, and social relations, might be more closely related to health outcomes than their absolute economic measures [18, 19].

This strand of research focused on social comparisons as important psychosocial pathways through which SES determine health [18], contributed to a growing interest in subjective measures of SES [17, 20–22]. Subjective social status (SSS) refers to the individual perception of relative position in the social hierarchy and is usually measured relative to others in the respondent’s close community [23].

Especially in the last two decades, SSS has been used in a variety of settings and shown to be associated, over and above objective SES markers, with different health outcomes [13, 24], such as self-reported health [21, 25, 26], mental health [22, 26], heart rate and sleep latency [27].

One of the explanations for SSS being a significant predictor of health is that it reflects the cognitive averaging of standard markers of the socioeconomic situation [28], that accounts for the past, current and future prospects and overall life chances [28–30], yielding a more precise measurement of overall SES [20].

The *MacArthur Scale of Subjective Social Status* (MacArthur Scale) [23] is one of the most widely used SSS measures in epidemiological studies. It has been used in some few African countries to study SES’s association with health-related outcomes [31–35]. However, as far as we know, there is no previous research using this approach to measure health inequalities in Central Africa countries, including Angola.

This subjective measure tool of SES might be particularly informative in settings like Dande, in Angola, where material and knowledge-related assets have little variation [36], and objective indicators such as income and occupation are challenging to measure due to greater reliance on the informal economy, self-employment, and seasonal activity [13, 37]. It may be easily accommodated in the context of Health and Demographic Surveillance System (HDSS), which typically perform routine visits to all households in a selected area, to provide a rapid assessment of a relevant social status construct, in addition to OSS indicators.

The effects of SES in mortality and women’s access to maternal health care have been previously studied in the HDSS population [36, 38].This study intends to explore health inequality determinants by testing SSS association with reported health and seeking help in formal health providers. To seek help in case of health care need reflects an action to prevent or treat illness [39]. The health-seeking behaviour (HSB) study traditionally relies on an individual-level approach, usually based on models of health behaviours, such as the Health Belief Model [40] and the Transtheoretical Model of Change [41]. Some of these theories are considered reductive because they give too much weight to individual’s behaviour as sole determinants of their health and little to the impact on individual health of environmental conditions that surround them, that are not controlled by individuals and, to a great extent, avoidable, such as a disadvantaged socioeconomic condition [17]. In this research, we sought to combine the individual process of health-seeking behaviour and the participants’ structural context by analysing their living conditions, social participation, OSS and SSS, to address health inequalities.

Therefore, our study has two aims. First, test the MacArthur Scale’s [23] application, and assess if it is a valuable tool to measure SES in the Dande HDSS area. Moreover, our analysis intends to comprehend how the MacArthur Scale relates to OSS conditions, and, finally, how it is associated with the surveyed population’s reported health and appropriate health-seeking behaviour (HSB)**.**

## Methods

### Study area

We have analysed data from the Dande HDSS, established in Dande Municipality, Bengo Province, located about 60 km to the north-east of Luanda, in Angola. The HDSS was implemented in 2009, and an initial census, performed between August 2009 and March 2010, registered the baseline population of 59,635 residents, distributed for 15,579 households. Since the initial census, update rounds (UR), consisting of periodic house-to-house visits, registered births, deaths, and migrations and collected information on household conditions, socioeconomic characteristics, and health-related issues.

The HDSS aims to provide relevant health, demographic, and socioeconomic data to inform local policies and research on the main diseases that affect the region [36, 42–44]. Detailed information about the HDSS has been published elsewhere [45].

### Data collection

The data collection was carried out during the 9th HDSS UR performed between January and August 2015, using structured questionnaires administered by trained fieldworkers who visited all the households of the demographic surveillance area (DSA). A total of 12,246 households were assessed and included in the study.

Data were collected via interviews, with the head of the household or an available household adult respondent. The questionnaire, launched after a pilot study, included routine questions collected in all HDSS URs, namely household and sociodemographic characteristics of the surveyed population, and a new section developed for the purposes of the study introducing subjective SES measures, health-related questions, media exposure and social capital of the participants (Supplementary File 1).

The study was designed with two main objectives: first, to test the application of the MacArthur Social Scale, a globally validated instrument in epidemiological research [27, 28, 31, 46], and assess the potential of this tool to measure SSS in the Dande HDSS population. Second, to understand associations between subjective and objective SES and the influence of SSS in reported health and HSB of the participants.

### Variables

#### Routine information collected

Place of residence, number of household residents, sociodemographic characteristics (sex, age, completed years of education), housing conditions (the existence of latrines and kitchen, number of rooms), drinking water source and the ownership of several assets in the household (as radio, television, freezer, car, and electricity).

The variable ‘Crowding’ was created to measure the number of residents per room. Overcrowding conditions were considered when there were more than three people per room [47].

The variable ‘Drinking water source’, was dichotomised based on the categories used by WHO/UNICEF [48]. Improved water corresponds to the sum of categories: piped into dwelling or yard, public tap, protected well, tanker truck, bottled water. Unimproved water corresponds to the sum of categories: unprotected well and open water sources located above ground, such as rivers, lakes, ponds, and irrigation channels.

#### New information collected

Household income, media exposure, participants’ social capital, the MacArthur Scale for measuring SSS, the use of mosquito bed nets, health care needs, and health-seeking behaviour.

Household monthly income was assessed and categorised in none or less than 10,000 Angolan Kwanza (AOA) monthly (equivalent to approximately 100 US Dollars in 2015), from 10,000 to 30,000 AOA and more than 30,000 AOA. The number of residents in the household receiving a fixed salary was asked, and its relationship with the total of people living in the household generated the variable ‘Proportion of residents with a fixed salary.’

Media utilisation was assessed through questions about whether household residents used to watch television, listen to the radio, and read newspapers.

Participants’ social capital was measured through respondents’ civic participation by asking them if they were part of a cultural, religious, and civic or sports collectivity/association (associative filiation). There is widespread interest in utilising social capital to understand the social process behind health inequalities, and evidence on his positive effect on health and HSB [49].

The MacArthur Scale of SSS [23] was administered to household respondents. It consisted of a symbolic ladder with ten rungs presented in a picture for which the following instruction was given: “Think of this ladder as representing where people stand in their communities – at the top of the ladder are the people who are best off, with more money, more education and who live better; at the bottom are those who are the worst off, who have the least money, least education and worse conditions. Where would you place your family on this ladder?”. Possible scores on the ladder range from 1 to 10, with higher scores indicating higher perceived social status. The scores of the ladder were grouped into quartiles.

The interview included questions about the existence of long-lasting insecticide net in each household, so as their number and usage (household members who slept under the bed net during the previous night). Those questions also measured health behaviours, given that Dande is an endemic malaria area, and the use of mosquito bed nets constitutes preventive behaviour [43].

#### Dependent variables

Two dependent variables were selected for the multivariate analysis. Participants were asked about any ill-health or injury among household residents within the month preceding the survey (yes/no), used as a proxy of the household health status. The first dependent variable, “**self-reported health care need**” was derived from these answers. Whenever a self-reported health care need was identified, participants were asked if they sought for help (yes/no), and where. This derived a second dependent variable “**appropriate health-seeking behaviour**”. Appropriate care in this study refers to the healthcare sought in formal health providers, such as health centres, hospitals, and private clinics, during illness episodes or any situation requiring medical attention (as opposed to those seeking for help from informal sources, namely traditional healers, church, market, pharmacy, nurses working at home, or family members).

The household’s geographical coordinates were collected using a geographical positioning system (GPS), and the distance between the households and the nearest health facility was measured based on the same system.

Depending on the date on which the interview took place, the corresponding season was indicated: the dry season, if data collection period occurred from May to September, or rainy season if it was from October to April.

### Statistical analysis

Chi-square tests and analysis of variance (ANOVA) were used to compare proportions and means, respectively, of demographic and household characteristics according to the quartiles of SSS distribution.

Receiver Operating Characteristic (ROC) Curves analysis was used to measure the discrimination capacity of the SSS ladder for the sociodemographic and household characteristics mentioned above.

The unadjusted association between self-reported health care needs (Outcome A) and had appropriate health-seeking behaviour (Outcome B), with several independent variables were measured using Odds Ratio (OR) and respective 95% Confidence Interval (CI). The *independent variables* included in the analysis were: SSS ladder, OSS indicators (household income, the proportion of residents with a fixed salary, years of schooling), the place of residence, household distance to a health facility, drinking water source, associative filiation, and bed net ownership.

In multivariate analysis, an exploratory model building approach was used, entering in block independent variables selected for being theoretically pertinent (based on literature review and previous analysis) to the outcome. The OR and the 95% CI were estimated using a binomial unconditional logistic regression.

The significant level was fixed in 0.05. All analyses were performed with Statistical Package for Social Sciences (SPSS) version 24.

## Results

Table 1 presents the households’ demographic and socioeconomic characteristics.

**Table 1:** Characteristics of the households

Variable (n)	Categories	n	(%)
**Place of residence** (*n* = 12,246)	Urban	9617	(78.5)
	Rural	2629	(21.5)
**Sex** (*n* = 12,128)	Male	4828	(39.8)
	Female	7300	(60.2)
**Age** (*n* = 11,040)	Mean ± sd min-max	38.41 ± 16.19 15–96	
**Years of schooling** (*n* = 10,753)	Mean ± sd min-max	4.78 ± 3.99 0–20	
**Nr. of household rooms** (*n* = 12,246)	Mean ± sd min-max	2.74 ± 1.34 1–13	
**Nr. of household residents** (*n* = 12,246)	Mean ± sd min-max	4.40 ± 2.70 1–20	
**Crowding** (*n* = 12,246)	Not overcrowding	11,154	(91.1)
	Overcrowding	1092	(8.9)
**Kitchen** (*n* = 12,246)	Yes	5167	(42.2)
	No	7079	(57.8)
**Latrine** (*n* = 12,245)	Yes	8709	(71.1)
	No	3563	(28.9)
**Do households own** (*n* = 12,242)	Electricity	8903	(72.7)
	Generator	1634	(13.3)
	Radio	6894	(56.3)
	Televison	8585	(70.1)
	Cell phone	9236	(75.4)
	Satellite dish	6753	(55.2)
	Fridge	1680	(13.7)
	Freezer	6219	(50.8)
	Car	1440	(11.8)
**Drinking water source** (*n* = 12,232)	Improved	6978	(57.2)
	Unimproved	5217	(42.8)
**Household members use to** (*n* = 12,246)	Watch television	9006	(73.5)
	Listen to the radio	5146	(42.0)
	Read newspaper	1395	(11.4)
**Associative filiation** (*n* = 12,246)	Yes	8111	(66.2)
	No	4133	(33.8)
**Distance to a health facility** (*n* = 12,246)	< 2 km	8302	(67.8)
	2–10 km	2450	(20.0)
	> 10 km	1494	(12.2)
**Household income** (*n* = 9485)	<= 10,000 AOA	2709	(28.6)
	10,001–30,000 AOA	5134	(54.1)
	> 30,000 AOA	1642	(3, 18)
**Nr. of residents with fixed salary** (*n* = 12,230)	Mean ± sd	0.51 ± 0.50	
	min-max	0–5	
**Proportion of residents with a fixed salary** (*n* = 12,230)	Mean ± sd	0.16 ± 0.25	
	min-max	0–3	
**SSS ladder** (*n* = 11,076)	Mean ± sd	2.91 ± 2.17	
	min-max	1–10	
**SSS quartiles** (*n* = 11,076)	1st quart. (1st step of the ladder)	3989	(36.0)
	2nd quart. (2nd step of the ladder)	2077	(18.8)
	3rd quart. (3rd to 4th steps of the ladder)	2754	(24.9)
	4th quart. (5th to 10th step of the ladder)	2256	(20.4)
**Someone in the household needed health care during the previous month** (*n* = 12,240)	No	6359	(52.0)
	Yes, and sought for help	5549	(45.3)
	Yes, but did not seek help	332	(2.7)
**Sought help** (*n* = 5549)	Informal sources	360	(6.5)
	Health care services	5189	(93.5)
**Bed net ownership** (*n* = 12,056)	Yes	3855	(32.0)
	No	8201	(68.0)
**Nr. of bed nets in the household** (*n* = 3855)	Mean ± sd	2.03 ± 1.24	
	min-max	0–12	
**Someone slept under a bed net during the previous night** (*n* = 3855)	Yes	2981	(77.3)
	No	874	(22.7)
**Season** (12,246)	Rainy season	5330	(43.5)
	Dry season	6916	(56.6)

Note: *sd* standard deviation; *min* minimum; *max* maximum; *AOA* Angolan Kwanza

*SSS* subjective social ladder.

From the households assessed 78.5% were in urban areas and 67.8% within a distance to a health facility lower than 2 km. The majority of the respondents were female (60.2%). The mean age of the participants in the study was 38.41 years and mostly had low levels of education (mean of 4.78 years of schooling).

Households had in mean 2.7 rooms (standard deviation, sd = 1.3), and 4.4 residents (sd = 2.7). We found overcrowding conditions in 8.9% of the households.

More than half of respondents declared owning electricity, radio, television, cell phone, satellite dish and freezer. The assets that respondents less declared to have in the household were a fridge (13.7%), a generator (13.3%), and a car (11.8%).

More than two thirds of the participants (66.2%) declared to have associative filiation.

In almost half of the households (48.8%), none of the residents had a fixed salary. The mean of the proportion of residents with a fixed salary per household was 0.16 (sd = 0.25).

For the SSS, the sample rated themselves on average below the midpoint of the scale (M = 2.91, sd = 2.17). The ladder’s quartiles showed a distribution of 36% of the households in the first quartile (1st of 10 steps of MacArthur ladder), 18.8% in the second quartile (2nd of 10 steps), 24.9% in the third quartile (3rd and 4th of 10 steps), and 20.4% in the fourth quartile (5th to the 10th steps of 10 steps).

Health care needs were reported by 48% of the respondents, of which 94.4% declared to have sought help. Of those who seek help, 93.5% did it in formal health services providers.

Thirty-two per cent of respondents declared owning at least one mosquito bed net in the household, among which 77.3% declared that someone in their household slept under the bed net during the previous night.

As shown in Table 2, the quartiles and the mean of the SSS ladder score differed significantly according to the demographic and socioeconomic characteristics.

**Table 2:** Bivariate analysis of Subjective Social Status ladder quartiles, by sociodemographic and household characteristics

Variable	1st quartile		2nd quartile		3rd quartile		4th quartile		***p-value***
Categories	n	(%)	n	(%)	n	(%)	n	(%)	
**Place of residence**									
Urban	2804	(31.6)	1647	(18.5)	2363	(26.6)	2072	(23.3)	< 0.001*
Rural	1185	(54.1)	430	(19.6)	391	(17.9)	184	(8.4)	
**Sex**									
Female	2256	(33.7)	1286	(19.2)	1684	(25.2)	1460	(21.8)	< 0.001*
Male	1690	(39.3)	773	(18.0)	1048	(24.4)	785	(18.3)	
**Age**									
Mean ± sd	40.09 ± 17.17		37.84 ± 16.16		36.93 ± 14.98		35.18 ± 13.52		< 0.001‡
**Completed years of schooling**									
Mean ± sd	4.44 ± 3.80		4.57 ± 3.77		5.00 ± 4.02		5.94 ± 4.32		< 0.001‡
**Nr. of household rooms**									
Mean ± sd	2.52 ± 1.22		2.76 ± 1.31		2.74 ± 1.36		3.26 ± 1.45		< 0.001‡
**Nr. of household residents**									
Mean ± sd	3.88 ± 2.56		4.52 ± 2.70		4.59 ± 2.68		5.36 ± 2.70		< 0.001‡
**Crowding**									
Not crowding	3668	(36.4)	1891	(18.7)	2455	(24.3)	2074	(20.6)	< 0.001*
Overcrowding	321	(32.5)	186	(18.8)	299	(30.3)	182	(18.4)	
**Kitchen**									
Yes	1433	(30.1)	913	(19.2)	1158	(24.3)	1257	(26.4)	< 0.001*
No	2556	(40.5)	1164	(18.4)	1596	(25.3)	999	(15.8)	
**Latrine**									
Yes	2576	(32.0)	1513	(18.8)	2073	(25.8)	1878	(23.4)	< 0.001*
No	1413	(46.6)	564	(18.6)	681	(22.4)	377	(12.4)	
**Do household members own**									
Electricity Yes	2535	(30.6)	1564	(18.9)	2158	(26.0)	2028	(24.5)	< 0.001*
No	1451	(52.1)	511	(18.3)	596	(21.4)	228	(8.2)	
Generator Yes	365	(24.3)	278	(18.5)	372	(24.7)	490	(32.6)	< 0.001*
No	3622	(37.9)	1799	(18.8)	2381	(24.9)	1766	(18.5)	
Radio Yes	1938	(30.6)	1155	(18.2)	1696	(26.8)	1544	(24.4)	< 0.001*
No	2050	(43.3)	922	(19.5)	1057	(22.3)	710	(15.0)	
Television Yes	2305	(28.8)	1520	(19.0)	2139	(26.7)	2045	(25.5)	< 0.001*
No	1683	(54.9)	556	(18.1)	615	(20.1)	210	(6.9)	
Cell phone Yes	2627	(30.5)	1605	(18.7)	2268	(26.4)	2104	(24.5)	< 0.001*
No	1361	(55.1)	472	(19.1)	486	(19.7)	150	(6.1)	
Satellite dish Yes	1656	(26.1)	1189	(18.7)	1679	(26.5)	1820	(28.7)	< 0.001*
No	2331	(49.3)	888	(18.8)	1073	(22.7)	435	(9.2)	
Fridge Yes	307	(19.3)	256	(16.1)	425	(26.7)	602	(37.9)	< 0.001*
No	3681	(38.8)	1820	(19.2)	2329	(24.6)	1650	(17.4)	
Freezer Yes	1526	(26.2)	1136	(19.5)	1464	(25.1)	1701	(29.2)	< 0.001*
No	2462	(47.0)	940	(17.9)	1288	(24.6)	552	(10.5)	
Car Yes	235	(17.1)	215	(15.7)	338	(24.6)	585	(42.6)	< 0.001*
No	3752	(38.7)	1862	(19.2)	2416	(24.9)	1671	(17.2)	
**Drinking water source**									
Improved	1901	(29.4)	1174	(18.2)	1817	(28.1)	1574	(24.3)	< 0.001*
Unimproved	2077	(45.5)	896	(19.6)	927	(20.3)	664	(14.5)	
**Household members use to**									
Watch television Yes	2517	(29.8)	1599	(18.9)	2231	(26.4)	2111	(25.0)	< 0.001*
No	1472	(56.2)	478	(18.3)	523	(20.0)	145	(5.5)	
Listen to the radio Yes	1453	(30.1)	774	(16.0)	1371	(28.4)	1236	(25.6)	< 0.001*
No	2536	(40.6)	1303	(20.9)	1383	(22.2)	1020	(16.3)	
Read newspaper Yes	194	(14.6)	191	(14.4)	398	(30.0)	543	(41.0)	< 0.001*
No	3795	(38.9)	1886	(19.3)	2356	(24.2)	1713	(17.6)	
Associative filiation									
Yes	2442	(32.3)	1314	(17.4)	1997	(26.4)	1802	(23.9)	< 0.001*
No	1546	(43.9)	762	(21.7)	757	(21.5)	454	(12.9)	
**Distance to the health facility**									
< 2 km	2469	(32.4)	1453	(19.1)	2018	(26.5)	1677	(22.0)	< 0.001*
2–10 km	806	(36.5)	396	(17.9)	509	(23.0)	500	(22.6)	
> 10 km	714	(57.2)	228	(18.3	227	(18.2)	79	(6.3)	
**Household income**									
< =10,000 AOA	996	(41.7)	366	(15.3)	695	(29.1)	330	(13.8)	
v10,001–30,000 AOA	1340	(27.9)	912	(19.0)	1304	(27.2)	1245	(25.9)	< 0.001*
> 30,000 AOA	494	(31.1)	200	(12.6)	369	(23.2)	526	(33.1)	
**Nr. of residents with a**									
**fixed salary** Mean ± sd	0.42 ± 0.56		0.62 ± 0.65		0.70 ± 0.68		0.93 ± 0.74		< 0.001‡
**Proportion of residents with a fixed salary** Mean ± sd	0.13 ± 0.23		0.17 ± 0.24		0.19 ± 0.27		0.22 ± 0.26		< 0.001‡
**Health care needs #**									
Yes	1783	(32.7)	1034	(19.0)	1417	(26.0)	1218	(22.3)	< 0.001*
No	2204	(39.2)	1042	(18.5)	1337	(23.8)	1036	(18.4)	
**If yes, did he/she** Yes	1697	(33.0)	981	(19.1)	1341	(26.1)	1127	(21.9)	0,014*
**sought for help** No	86	(28.1)	53	(17.3)	76	(24.8)	91	(29.7)	
**Sought for help**									
Informal sources	129	(41.3)	59	(18.9)	81	(26.0)	43	(13.8)	0.001*
Health care services	1568	(32.4)	922	(19.1)	1260	(26.1)	1084	(22.4)	
**Bed net ownership**									
Yes	1318	(37.5)	651	(18.5)	826	(23.5)	721	(20.5)	0,035*
No	2593	(35.1)	1397	(18.9)	1898	(25.7)	1507	(20.4)	
**Nr. of bednets in the household**									
Mean ± sd	1.87 ± 1.12		1.95 ± 1.06		2.12 ± 1.29		2.39 ± 1.43		< 0.001‡
**Slept under a bednet ##**									
Yes	1065	(39.0)	484	(17.7)	651	(23.8)	533	(19.5)	0,001*
No	2846	(34.8)	1564	(19.1)	2073	(25.3)	1695	(20.7)	
**Season of the interview**									
Rainy	1577	(33.9)	864	(18.6)	1104	(23.8)	1102	(23.7)	0,001*
Dry	2412	(37.5)	1213	(18.9)	1650	(25.7)	1154	(17.9)	

Note: * *χ*^2^

‡ ANOVA.

# Someone in the household needed health care during the previous month.

## Someone slept under a bed net during the previous night.

The respondents’ mean age decreased from the 1st quartile (40.09 years, sd = 17.18) to the 4th one (35.19, sd = 13.53).

Education followed a different pattern and increased along with the quartiles of the ladder. The mean education in schooling years was 4.44 (sd = 3.80) in the 1st quartile and 5.94 (sd = 4.32) in the 4th quartile.

There were statistically significant associations between SSS ladder quartiles and the number of rooms, residents in the household, the number of residents with a fixed salary, and the proportion of residents with a fixed salary per household (*p* < 0.001). In each of those variables, the mean increased from the 1st to the 4th quartile of the SSS ladder.

Residents living in households in rural areas, in overcrowding conditions, consuming unimproved water, without a kitchen, latrine, and deprived of electricity, television, cell phone, satellite dish, or freezer, were less frequent in the 4th SSS quartile.

The results showed that people who do not use to have contacts with media (television, radio, or newspapers), were mainly positioned in the 1st quartile of SSS ladder. Those who affirmed to have the habit of reading the newspaper were mostly in the 4th quartile of the ladder.

The distance of the household to a health facility was strongly associated with the quartiles of the ladder (*p* < 0.001) with those living more than 10 km from a health facility, more represented in the 1st quartile.

Households where the declared income was less than 10,000 AOA, were mainly placed in the 1st quartile of the SSS ladder (41.7%).

We also found an association between the ladder quartiles and the reporting of health care needs (*p* < 0.001), as well as having sought for help (*p* = 0.014). Individuals whose HSB was from informal sources were mainly positioned in the 1st quartile of the SSS ladder (41.3%).

A statistically significant association between ladder quartiles and bed net ownership (*p* = 0.035), and utilisation (*p* < 0.001) was found. The mean number of bed nets in the household increased from the 1st (1.87, sd = 1.12), to the 4th quartile (2.39, sd = 1.43) of the SSS ladder.

Considering the significant association between the quartiles of the SSS ladder and almost all sociodemographic and economic characteristics, we tested the capacity of the ladder to discriminate each variable through the ROC Curves analysis (Table 3). The results showed low AUC (Area Under the Curve) values, demonstrating the ladder’s weak ability to predict the household characteristics of the surveyed population. Table 3 describes the responsiveness of the Mac Arthurs’ SSS scale in terms of sensitivity and specificity for detecting changes in covariates, namely discriminating those who have and those who do not have certain socioeconomic characteristics.

**Table 3:** Diagnostic value from ladder according to Objective Socioeconomic Status indicators

	Cutoff point	Sensitivity	Specificity	Youden Index ^c^	AUC^a^	95% CI
**Place of residence** (urban)	2.5	0.50	0.74	0.24	0.651	0.639–0.663
**Sex** (female)	1.5	0.66	0.39	0.06	0.532	0.521–0.543
**Education**						
No education^b^	9.5	**0.98**	0.02	0.001	0.549	0.536–0.562
Primary (1–6)^b^	2.5	0.52	0.45	−0.03	0.519	0.507–0.530
Secondary (7–13)	2.5	0.52	0.56	0.08	0.551	0.539–0.564
Tertiary (University)	3.5	0.59	0.68	0.26	0.649	0.605–0.693
**Overcrowding**	2.5	0.49	0.55	0.04	0.517	0.499–0.536
**Residents with a fixed salary**						
None	9.5	**0.98**	0.01	−0.02	0.642	0.631–0.652
One	1.5	0.72	0.42	0.14	0.582	0.571–0.593
Two or more	2.5	0.72	0.57	**0.29**	**0.693**	0.676–0.710
**Household income**						
< = 10,000 AOA^b^	9.5	**0.98**	0.01	−0.01	0.594	0.581–0.607
10,001–30,000 AOA	1.5	0.72	0.38	0.10	0.547	0.534–0.559
> 30,000 AOA	4.5	0.33	0.78	0.11	0.548	0.531–0.564
**Improved water source**	2.5	0.52	0.65	0.18	0.604	0.593–0.615
**Kitchen**	4.5	0.26	0.84	0.11	0.574	0.563–0.585
**Latrine**	1.5	0.68	0.47	0.15	0.595	0.584–0.607
**Watch television**	1.5	0.70	0.56	0.26	0.677	0.666–0.688
**Listen to the radio**	2.5	0.54	0.62	0.15	0.586	0.573–0.597
**Read newspaper**	2.5	0.71	0.58	**0.29**	0.686	0.671–0.701
**Associative filiation**	2.5	0.50	0.66	0.16	0.596	0.585–0.607
**Electricity**	1.5	0.69	0.52	0.22	0.644	0.633–0.655
**Generator**	3.5	0.44	0.70	0.14	0.603	0.587–0.618
**Radio**	2.5	0.51	0.63	0.14	0.587	0.577–0.598
**Television**	1.5	0.71	0.55	0.26	0.671	0.661–0.682
**Cell phone**	2.5	0.51	0.74	0.25	0.666	0.655–0.677
**Satellite dish**	2.5	0.55	0.68	0.23	0.660	0.650–0.670
**Fridge**	2.5	0.65	0.58	0.23	0.652	0.637–0.667
**Freezer**	1.5	0.74	0.47	0.21	0.642	0.632–0.652
**Car**	3.5	0.55	0.72	0.26	0.675	0.658–0.689
**Bed net ownership**	7.5	0.05	**0.96**	0.01	0.489	0.478–0.501
**Season (Rainy)**	5.5	0.16	0.90	0.06	0.534	0.524–0.545

Case: yes|true; Control:no|false.

^a^ *AUC* Area under the curve.

^b^Inverted the score

^c^ Youden Index [50].

The variables that the ladder better discriminates in terms of sensitivity are the traditional OSS indicators, namely the lower categories of education, household income, and the number of residents with a fixed salary per household (Sen = 0.98). The ladder is very specific for the discrimination of bed net ownership (Spe = 0.96).

The values of AUC close to 0.5 showed that the MacArthur Scale was not a good measure of separability regarding the selected economic conditions of the population in the DSA. The higher AUC values found in our results were 0.693, 0.686, 0.677, and 0.675, referring to a 68 to 70% chance of the ladder to distinguish, respectively, between those living in households with none, one or two/more residents with a fixed salary, those with or without the habit of reading the newspaper and watch television, and those with or without a car.

Table 4 analyses how SSS and OSS measures predict a change in self-reported health care needs (Outcome A) and appropriate HSB (Outcome B).

**Table 4:** Adjusted and unadjusted Odds Ratio (OR) of reported health care need (Outcome A), and appropriate health-seeking behaviour (Outcome B), against sociodemographic variables, from binomial logistic regression

Variables	Outcome A					Outcome B				
	n	Unadjusted OR	***p***	Adjusted OR^a^	***p***	n	Unadjusted OR	***p***	Adjusted OR^a^	p
**SSS quartiles**	11,071					5452				
1st quartile	3987	1		1		1173	1		1	
2nd quartile	2076	1.23 (1.10–1.36)	< 0.001	1.36 (1.18–1.56)	< 0.001	1034	1.29 (0.93–1.77)	0.122	1.52 (1.05–2.19)	0.025
3rd quartile	2754	1.31 (1.19–1.44)	< 0.001	1.10 (0.98–1.24)	0.120	1417	1.28 (0.96–1.71)	0.093	1.74 (1.24–2.43)	0.001
4th quartile	2254	1.45 (1.31–1.61)	< 0.001	1.12 (0.99–1.28)	0.067	1218	2.07 (1.46–2.95)	< 0.001	2.23 (1.52–3.26)	< 0.001
**Household income**	9840					4730				
<= 10,000 AOA	2709	1		1		1222	1		1	
10,001–30,000 AOA	5131	1.17 (1.06–1.28)	0.001	0.96 (0.86–1.08)	0.540	2510	1.66 (1.27–2.17)	< 0.001	1.33 (0.97–1.82)	0.074
> 30,000 AOA	1640	1.89 (1.67–2.14)	< 0.001	1.56 (1.34–1.81)	< 0.001	998	1.52 (1.09–2.12)	0.014	1.09 (0.74–1.61)	0.663
**Proportion res/fixed salary**	12,224/12,246	0.76 (0.66–0.88)	< 0.001	0.53 (0.43–0.64)	< 0.001	5870/5881	1.87 (1.06–3.32)	0.032	1.20 (0.63–2.29)	0.586
**Years of Schooling**	10,747/12,246	1.00 (0.99–1.01)	0.007	0.98 (0.97–0.99)	0.003	5368/5881	1.02 (0.99–1.05)	0.129	0.99 (0.96–1.03)	0.673
**Place of residence**	12,240					5881				
Rural	2628	1		1		871	1		1	
Urban	9612	2.20 (2.01–2.40)	< 0.001	1.57 (1.30–1.88)	< 0.001	5010	1.60 (1.23–2.09)	< 0.001	0.73 (0.40–1.33)	0.307
**Distance to health facilities**	12,246					5881				
< 2 km	8302	1		1		4196	1		1	
2–10 km	2450	0.92 (0.84–1.01)	0.075	1.05 (0.92–1.19)	0.491	1187	1.52 (1.11–2.08)	0.010	1.54 (1.03–2.29)	0.04
> 10 km	1494	0.49 (0.44–0.55)	< 0.001	0.66 (0.52–0.83)	0.001	498	0.51 (0.38–0.70)	< 0.001	0.37 (0.18–0.73)	0.005
**Drinking water**	12,189					5228				
Unimproved	5212	1		1		2015	1		1	
Improved	6977	1.55 (1.44–1.66)	< 0.001	1.15 (1.03–1.30)	0.016	3513	1.46 (1.18–1.81)	0.001	1.65 (1.20–2.26)	0.002
**Associative filiation**	12,239					5881				
No	8111	1		1		4324	1		1	
Yes	4128	1.89 (1.75–2.04)	< 0.001	1.17 (1.05–1.29)	0.004	1557	1.39 (1.10–1.74)	0.005	1.43 (1.08–1.89)	0.013
**Bed net ownership**	12,051					5788				
No	3854	1		1		3691	1		1	
Yes	8197	1.46 (1.35–1.57)	< 0.001	1.52 (1.38–1.68)	< 0.001	2097	1.35 (1.07–1.71)	0.011	1.46 (1.10–1.95)	0.009

Note: Values of OR > 1 indicate increased risk of self-reported health care need and odds of appropriate HSB. ^a^ Adjusted for all variables listed

In the bivariate analysis, all the selected explanatory variables, were significantly associated with Outcome A, and most associations remained significant after adjusting for the presence of covariates.

Results from the adjusted model show that both objective and subjective measures of SES predicted the socioeconomic patterning of health care needs differently. The residents who place themselves in the 2nd quartile of the social ladder had greater odds of reporting health care needs (Adjusted OR = 1.36, 95% CI = 1.18–1.56), compared with those at the bottom of the scale. A higher income also increased the odds of health care need report (OR = 1.56, 95% CI = 1.34–1.81). On the contrary, having more years of schooling and a higher proportion of residents with a fixed salary per household decreased the likelihood of reporting a health problem within the household.

Respondents living in urban areas, drinking improved water, with associative filiation, and owning bed nets, were more likely to affirm the existence of health problems in their households. In contrast, those living furthest from health facilities reported less health care needs.

Regarding Outcome B, the bivariate analyses indicate that subjective and objective measures of SES (except years of schooling) were independently associated with appropriate HSB.

In the fully adjusted model, the OSS markers lose statistical significance. The SSS remained statistically significant. Respondents at the top of the ladder were 2.23 (95% CI = 1.52–3.28) more likely to have an appropriate HSB than those at the bottom, while greater distances to health facilities decreased the likelihood of choosing an appropriate health care provider (Adjusted OR = 0.37, 95% CI = 0.18–0.73).

## Discussion

We have used data from the Dande HDSS to test the application of the MacArthur Scale as a measure of SSS, to understand how it relates with OSS indicators and to analyse the relationship with reported health need and appropriate HSB. Both health outcomes were positively associated with SSS, independently of OSS.

In general, the respondents were able to understand the question of the MacArthur Scale and to provide valid answers. The ladder is usually considered a streamlined and effective measure of social status, as it offers a viable and easier alternative to traditional SES measures, which require more exhaustive questions and may suffer from reporting issues or bias [23, 51].

The income, for example, is one of those typical SES indicators more susceptible to bias, and high rates of non-response [52]. In various LMIC, including Angola, many people do not know or do not want to report their income, and measuring it may be challenging, given the weight of informal sector activities, the revenue fluctuations, and the remittances, that are difficult to quantify. Subsistence agricultural activities are also often not considered [53].

Turrel [54] summarises the results of the non-response rates observed in other settings for income data, that range from 10 to 25%. In our study, the comparison between rates of non-response to the MacArthur ladder (10%) and income questions (44% on the exact amount of revenues and 23% in the question organised in categories), corroborates the idea that the collection of income data is problematic [46, 52, 54].

Additionally, the results suggest that the SSS in the Dande HDSS population follows a socioeconomic position gradient distribution obtained with OSS indicators, reassuring its utility [46, 55]. Though SSS was, in part, designed as an attractive and more encompassing alternative to traditional measures, its use still intends to tap objective socioeconomic variation [51].

The perceived perceptions of SES, significantly associated with almost all surveyed sociodemographic and household characteristics, indicate a relative homogeneity of the population. The mean ranking of SSS using a ten rungs ladder, was 2.91 (sd = 2.17), with 79.7% of respondents placing themselves on rungs one through four, resulting in a distribution of the ladder strongly skewed to the lower rungs of the scale. At the best of our knowledge, the mean ranking in the DSA is only similar to the results obtained in a study conducted in rural Ethiopia (M = 2.9, sd = 1.3) [32]. Further research using the MacArthur Scale reported higher and more symmetric SSS mean scores [56].

The concentration of responses on the lower rungs of the ladder can have different explanations. First, the context from which our sample was drawn. Angola is a developing country, ranked number 149 out of 189 countries in the Human Development Index [57], a position that involves poor scores in terms of education and health indicators [37]. Since the end of 2014, the country is suffering an economic crisis resultant of the slide in the selling price of crude oil, its main export. In 2015, 29.4% of the national population lived below the poverty line of $1.9/day, and 53.9% lived with less than $3.1/day [58]. In our sample, a gross estimate (calculated using the higher interval of household income divided by the number of residents per household) of the population living in those conditions was, at least, 42.2 and 63.5%, respectively. It is possible that the structural/ material conditions under which people live, make them feel deprived of options for upward social mobility [37], and the cycle of disadvantages, including uncertainty, material constraints and/or a feeling of fewer opportunities, influences an almost general low assessment in the subjective social scale [19, 59]. According to different authors, someone’s placement in the social ladder appears primarily to involve cognitive averaging of standard markers of socioeconomic position along with their assessment of past, current, future prospects, and overall life chances [25, 28–30].

An alternative hypothesis is that the low assessments of social position result from the expected convergence between OSS and SSS. Low educational levels and economic vulnerability characterise the population in our study. In a sample of 12,246 respondents, with a mean age of 38.41 years, 23.7% had no school qualifications, 45.7% primary education, 29.0% secondary education, and only 1.6% had tertiary education. In almost half of the households, no one received a fixed salary, and the average proportion of residents with a fixed salary per household was far from one (M = 0.16, sd = 0.25). This scenario suggests a high weight of informal economic activities and financial insecurity due to fluctuating incomes. In a study conducted the Angolan neighbourhoods of Luanda and Kalandula, 32.4 and 13.7% heads of the households declared informal employment, respectively, and 1.9 and 69.9% had farming as the main occupation. Those households were vulnerable to sudden shocks, e.g*.*, reduced access to food and income, increases in expenses for health and education, and the death or absence of main breadwinners [37]. Previous studies demonstrated that SSS was determined by aspects such as education, household income, and also feeling of financial security regarding the future [28].

In developed settings, respondents to the MacArthur ladder scale have primarily valued material wealth, occupational status, and education in providing their self-perceptions of social status [23, 27]. Nevertheless, the SSS may capture the influence of several other social and psychological variables associated with the relative position in society distinctively relevant to overall health. SSS may perform similarly to measures of self-rated health: capture the influence of variables beyond objectively measured health risk factors, allowing it to be one of the most reliable mortality predictors, worldwide [60, 61].

The SSS ladder provides a collective measure of social status. Respondents are usually asked to consider different aspects of their relative status (as opposed to focusing on specific asset ownership, education or occupation), and thus may attribute different weights to the distinct components of their socioeconomic position [23]. SSS should be distinguished from other SES measures since it is purported to be a different construct [62, 63], which might explain the weak capacity of the ladder to discriminate several objective economic conditions in our study. Although related, objective indicators and subjective rankings capture different aspects of social standing [9, 25].

People may have a deeper understanding of the meaning of their position on a given aspect of the social structure that is specific to their context, and this may be particularly useful in rural developing African settings. Even in a small area where the majority of the houses are made of clay, with a tin roof, and most people share a bathroom, there is still someone that places him/herself in a higher social hierarchical position, based on culturally-specific values [21]. Such values can be related to television ownership, with the ability to read the newspaper, with the position held in the church, with one’s ability to use traditional healing procedures, or even with the number of wives and children, considered a sign of manhood and fertility. These features may thus contribute to a specific ‘cognitive averaging’ of standard dimensions of SES, as previously suggested [55, 64]. A qualitative approach to such representations in this setting would be meaningfully valued.

Our results support previous findings of an association between SES indicators and health-related issues and showed that SSS influence health needs reporting and health-seeking behaviour above and beyond traditional SES indicators [65–67].

It is important to note that our study’s health status question referred to all household members and that the reported health needs were not necessarily felt by the respondents who positioned the household on the SSS. However, in agreement with the literature, we found that SES’s objective and subjective measures were associated with reported health when entered separately and that the SSS remains significant after controlling for OSS indicators [25, 27, 28, 68]. Although, our findings differ from those observed in some research that found that perception of lower status was associated with poor reported health [20, 52].

In Dande, as in other developing settings in Ghana, Guinea, and Tanzania, the health reported needs were more common among the better-off than in the most deprived households [69–71]. Individuals standing in the 2nd quartile of the ladder and with higher income had more odds of reporting illness in their households than those in the bottom of the ladder and with lower incomes. Living in urban areas, having access to improved water, social participation, and bed net ownership also increased the odds of reporting health care needs.

Perhaps that is because the socioeconomic environment influences the concepts of illness, and better-off households are more likely to recognise their signs [69]. More deprived people may perceive illness as a normal life feature and do not consider it an event worth reporting [70]. Furthermore, they might tend to ignore illness, given the costs that being sick imply, such as treatment costs or work absence [71, 72]. Unequal access to health care services, or differences in environmental conditions may lead to divergent health and morbidity experiences resulting in different self-reported health [71].

Considering the reported health status, we analysed the HSB of the participants, i.e.*,* any action or inaction of those who perceive to have a health problem, themselves or within the household, for the purpose of finding an appropriate remedy [39]. The appropriate HSB was defined as consulting a qualified medical professional or seeking healthcare in a formal health care provider [39].

In our sample, 93.5% of the individuals that reported care needs and sought for help, used formal health care services, and 6.5% relied on informal sources, proportions identical to those found in the national population, of 93.4 and 6.6%, respectively [73]. The utilisation of health care services is considerably higher than that found in studies conducted in rural areas of Nigeria and Kenia [74, 75] and similar to results in South Africa [76]. Distance is a known barrier to health care utilization, as it is linked to lack of transport, poor access and costs [77]. Therefore, the high proportion of respondents with appropriate HSB might be explained partly by the fact that in our study, 78.5% of the participants were living in urban areas and 67.8% at distances lower than 2 km from health facilities.

The SSS showed to be a factor affecting the respondent’s choice of health care provider, given that people in the highest quartiles of the ladder were twice more likely to seek help in formal health services than those at the bottom of the ladder. The significant effect of SSS on appropriate HSB remained even after controlling for the objective SES markers [20], suggesting that a sense of social ordering is more important for health behaviors than income.

As expected and previously documented, the odds of appropriate HSB decreased with increasing distance of residences to health facilities [78–80], which suggests the need to still work on the main health care access barriers affecting this setting.

The improved water consumption, which in most of the cases in the households of the DSA imply the treatment of drinking water, as well as the ownership of bednet in this endemic malaria area [43] also constitute preventive health attitudes, and both variables remained significant in the adjusted model to determine appropriate HSB.

The social capital, measured through civic participation, defined as any associative affiliation, was also a predictive variable of HSB. It explores individuals’ inter-relationships within social systems, cultural norms and system constraints, and interprets their behaviours as a product of these relationships rather than something exclusively intrinsic to the individual [4, 49].

### Strengths and limitations

To our knowledge, this is the first study testing the MacArthur Scale, and that uses subjective measures to capture SES in Angola. SSS is an increasingly utilised measure in social and epidemiological studies, and there are several advantages for using it instead or complementing objective measures of SES. The MacArthur Scale is of ease use and was developed to capture the common sense of social status based on usual SES indicators [81]. The abstract structure of the question facilitates comparisons between studies conducted in different populations [20] and its use in research worldwide [22, 29, 46, 66] had proved the association between SSS and several health outcomes, usually over and above the influence of OSS measures [20, 81]. Therefore, this study contributes to the literature on health determinants in Angola by introducing a more robust indicator of SES.

It is noteworthy that this study has some limitations. The data regarding health is based exclusively on self-reported data. Future investigation should assess participants’ current health objectively, to understand the impact of SES, and, in particular, SSS, in the health of the population. Another limitation is the homogeneity of the sampled population. The use of SSS measures with proximal referent groups may not fully capture the impact of the perceived hierarchical rank [21], since that the Dande population may share similar cultural and social ways of living, reflected on health and illness experiences [71]. Further research with a more heterogeneous population in Angola is needed to understand how the effects of SES reflect across health outcomes, i.e., greater social and cultural variation may produce a higher level of inequality in reported health needs and health-seeking behaviour.

Finally, it is worth noting that the data used in this research were collected in 2015, so the patterns and trends in the relationship between objective and subjective measures of SES, the reported health needs, and the HSB may have changed. However, this does not alter the viability of using the MacArthur Scale as an appropriate tool to measure SSS in future research to assess its influence on health outcomes.

## Conclusions

SSS may be an important indicator considering the addition it provides to SES assessment and for the study of health inequalities. This may be particularly relevant in Angola, where society experienced rapid socioeconomic and structural changes, but huge disparities exist within the country concerning income, opportunities, human capital, access to health care services, and health outcomes [82]. Since the end of the civil war (2002), Angola has gone through a robust economic growth but not followed closely by an improvement in several social indicators [83], and very scarce evidence exists documenting and contextualising the health status of the population, which is of great relevance for health policy.

The results of this study suggest that SSS may be a useful, feasible and valuable assessment tool in this developing setting, capturing social status perceptions otherwise unreachable by traditional SES measures, relevant for the study of socioeconomic disparities, and that might translate into health inequalities.

Further research should explore associations between objective health outcomes, such as morbidity and mortality, and SSS. Additionally, it would be interesting to use longitudinal data to understand the extent to which SSS is modifiable over time and integrates past, present experiences and future prospects, and how those impact health outcomes. Both proposals are feasible within the scope of HDSS activities.

## Supplementary Information

**Additional file 1: Supplementary File 1.** Questionnaire (English version)

## Data Availability

The anonymised data set is freely available in Zenodo database (10.5281/zenodo.4707929).

## Abbreviations

AOA: Angolan kwanza
AUC: Area under the curve
CI: Confidence interval
DSA: Demographic surveillance area
GPS: Geographical positioning system
HDSS: Health and demographic surveillance system
LMIC: Low and middle-income countries
MacArthur Scale: MacArthur scale of subjective social status
OR: Odds ratio
OSS: Objective socioeconomic status
ROC: Receiver operating characteristic
Sd: Standard deviation
Sen: Sensitivity
SES: Socioeconomic status
Spe: Specificity
SSS: Subjective social status
UR: Update rounds
