# Supplementary material for: Examining the relation between the subjective and objective social status with health reported needs and health-seeking behaviour in Dande, Angola
Source: BMC Public Health. 2021 May 25;21:979. doi: 10.1186/s12889-021-11003-4 (PMC8152355; doi:10.1186/s12889-021-11003-4)
Supplement: Supplementary file 1 — Additional file 1: Supplementary File 1. Questionnaire (English version) [file 12889_2021_11003_MOESM1_ESM.pdf]

Supplementary File 1 – Questionnaire (English version)

HDSS – Model of the household questionnaire used in the 9th update round.

Date of interview: **Fieldworker:**

Name of the Head of the household:

Housing characteristics

**Walls's material**
Adobe
Brick/bloc
Wattle and daub
Woven straw
Iron sheet
Wood
Other. What

**Roof**
Straw
Iron sheet
Roofing shingles
Fibrocement
Other. What

**Floor**
Concrete / Cement
Crude floor (cement)
Ceramic tiles
Earth
Carpet
Other. What

**Number of rooms**	

**The house has kitchen**	**Yes/No**

**Drinking water source**
Public tap or standpipe
Piped into dwelling or yard
River
Irrigation channel
Lagoon
Well and open sources above ground
Borehole
Water tank
Other

**Treat drinking water**
Yes/no
**If yes, what use for treating water.**
Leaches
Ume stone
Boils
Filter
Other method

**Bathing water source**
Public tap or standpipe
Piped into dwelling or yard
River
Irrigation channel
Lagoon
Well and open sources above ground
Borehole
Water tank
Other

**Main cooking fuel**
Firewood
Charcoal
Kerosene
Gas
Electricity
Other

**Household assets**
Electricity
Generator
Radio
Television
Cell phone
Satellite dish
Fridge
Freezer

**The house has latrine**
Yes/No

**If yes (with latrine)**
With water
Without water

**If yes (with latrine)**
Just for family
Shared with neighbours

**Transports in the household**
Car
Motorcycle
Bicycle
Other. What

**Livestock Breeding**
Yes/No
**What kind of animals**

Household income and Social Status Subjective measures

Monthly income (net)
____

**Monthly income (categories)**
None
Less than 10.000 AOA
Between 10.001 and 30.000 AOA
Between 30.001 and 50.000 AOA
Between 50.001 and 70.000 AOA
More than 70.000 AOA
Do not know/ Do not answer

**How many people in the household receive a fixed salary**
No one
1 person
2 persons
3 persons
4 persons
5 or more persons
Do not know/ Do not answer

Think of this ladder as representing where people stand in their communities – at the top of the ladder are the people who are best off, with more money, more education and who live better; at the bottom are those who are the worst off, who have the least money, least education and worse conditions. Where would you place your family on this ladder?". [Possible scores on the ladder range from 1 to 10, with higher scores indicating higher perceived social status]. **Note** The ladder was shown to respondents in A4 size.

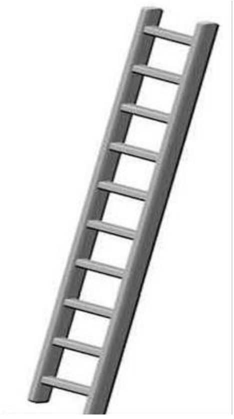

Media exposure and Social Capital

**Someone in the household uses to watch television, listen the radio, read newspapers? Yes/No**	
Television	
Radio	
Newspaper	
Other	

**Someone in the household belongs to some kind of collectivity. Yes/No**
Religious
Cultural
Sportif
Civic. Other

Health cares need and Health seeking behaviour

**Any ill-health or injury among household residents within the month preceding the survey (yes/no)**

**If yes, sought help?**

**If sought for help, where**	**If no, why**
Church	The problems disappeared
Family or neighbhours	It is expensive / No money
Market or pharmacy	It is far from home
Heath facility	Too full / time consuming
Traditional healers	Other
Nurses working at home	
